# Interactive Effects of Methionine and Lead Intake on Cognitive Function among Chinese Adults

**DOI:** 10.3390/nu14214561

**Published:** 2022-10-29

**Authors:** Xiaomin Sun, Zhongying Li, Yingxin Chen, Tao Xu, Jing Shu, Lin Shi, Zumin Shi

**Affiliations:** 1 Global Health Institute, School of Public Health, Xi’an Jiaotong University Health Science Center, Xi’an 710061, China; 2 School of Food Engineering and Nutritional Science, Shaanxi Normal University, Xi’an 710119, China; 3 Human Nutrition Department, College of Health Sciences, QU Health, Qatar University, Doha 2713, Qatar

**Keywords:** animal methionine, plant methionine, lead intake, cognitive function, interactive effect

## Abstract

The association between methionine intake and cognitive function is inconclusive. We aimed to assess the association between methionine intake and cognitive function in Chinese adults and to explore the interaction between methionine and lead intake. Data from 4852 adults aged ≥55 years from the China Health and Nutrition Survey were used. Cognitive function was measured in 1997, 2000, 2004, and 2006. A 3-day, 24-hour recall was used to assess methionine and lead intake from different protein sources. Multivariable mixed linear regression was used in the analyses. Total methionine intake was positively correlated with cognition. There was a significant interaction between animal methionine and lead intakes. In subgroup analyses, across the quartiles of animal methionine intake, the regression coefficients (95% CI) for global cognition were 0.00, 0.57 (0.17 to 0.98), 1.18 (0.73 to 1.62), and 1.80 (1.31 to 2.29), respectively, while they were 0.00, −0.73 (−1.12 to −0.34), −0.83 (−1.26 to −0.41), and −1.72 (−2.22 to −1.22) across the quartiles of plant methionine intake, respectivelyThe association between animal methionine intake and cognition was stronger among adults with a low lead intake. In conclusion, animal methionine and plant methionine intake were positively and inversely associated with cognition, respectively. Lead intake modified the association between animal methionine intake and cognition.

## 1. Introduction

Cognitive impairment is a major and growing global health challenge. The number of people with dementia—a severe cognitive impairment—is predicted to triple from 55 million in 2021 to 139 million in 2050, with over 60% living in low- and middle-income countries [1]. Population aging and the growing frequency of cognitive impairment pose a heavy burden on healthcare systems. China has the largest population with dementia in the world and is one of the fastest-aging societies [2]. According to the seventh Chinese national census of 2020, the number of individuals aged 60 years and above was 264 million, accounting for 18.7% of the population [3]. In addition to aging, socioeconomic factors (e.g., education, income), lifestyle factors (including smoking and physical activity), and chronic conditions (e.g., traumatic brain injury, thyroid function, COPD) have been found to be associated with cognition [4,5,6,7,8,9]. As most types of cognitive impairment are incurable, investigations of potentially modifiable lifestyle and environmental risk factors to prevent cognitive decline are urgently needed.

Methionine is a nutritionally indispensable sulfur amino acid (SAA) that is involved in creatine synthesis and nearly all methylation reactions in vivo via the transmethylation reaction [10]. Recently, methionine restriction has been suggested to exert protective effects against age-related cognitive decline via several pathways in rat brains, e.g., changing the lipid composition and alleviating neuroinflammation and oxidative stress [11,12]. On the other hand, methionine supplementation has been demonstrated to improve cognition in folate-deficient rat models [13]. Additionally, methionine treatment could promote resilience to chronic stress through an epigenetic mechanism (i.e., increasing histone methyltransferase and inhibiting the levels of histone H3 lysine (K9) trimethylation) [14]. Consistently, some evidence from investigations on human tissues has also shown that high methionine levels could protect the brain from damage [15,16]. Nevertheless, compared with accumulating evidence in laboratory animal models, results from population studies on methionine intake and cognition are rare. In a population-based study of 4457 individuals aged 60 years and above, high intake of methionine cycle metabolites (i.e., serum folate and homocysteine) were found to be related to mild cognitive impairment (MCI) [17]. Folate-responsive dietary patterns were positively associated with MCI, while homocysteine-responsive patterns were inversely associated with MCI [17]. A prospective cohort including 15,083 US adults with 16.9 years of follow-up reported that adults with the highest quartile of methionine intake (Q5) were 2.5 times more likely to have a diabetes-caused death than Q1, while the associations became nonsignificant when further adjusted for animal and plant protein [18]. Previously, methionine was mainly derived from plant-based foods (e.g., soybeans and peas), while more methionine provided by animal-based foods (e.g., meat, fish, milk, and eggs) is now entering the human food supply than at any time in history [19]. Herein, the investigations are focused on effects of methionine intake—especially source-specific intake—on cognition in adults.

Many environmental chemicals have long been known to be neurotoxic; among them, the role of lead is of particular interest, given its low excretion and common accumulation [20]. In China, contamination of food with heavy metals (e.g., lead, cadmium, and arsenic) has been a major public concern due to environmental pollution [21]. A recent review reported that low-level lead exposure increases the risk of brain abnormalities, cognitive decline, and degenerative disease [22]. A prospective population-representative birth cohort study found that childhood lead exposure was associated with lower cognitive function in adulthood [23]. A sub-cohort of the Nurses’ Health Study also reported that lead exposure in adulthood is associated with impaired cognitive decline in later life [24]. Lead exposure is known to affect cognitive function by selectively altering the N-methyl-D-aspartate receptor (NMDAR) activity [25]. On the other hand, methionine treatment was suggested to improve cognitive function in lead-exposed rats, and this may be attributable to the recovery of deficits in NMDAR mRNA and protein expression in the hippocampus [26,27]. It was also reported that methionine treatment could help decrease the burden of lead in blood and tissues and increase its fecal excretion in rats [28]. Together, existing evidence suggests that the association between dietary methionine and cognitive function may be significantly affected by dietary lead intake. However, no large-population study has examined this interaction.

To fill these research gaps, we analyzed the longitudinal survey data from the China Health and Nutrition Survey (CHNS) and aimed to assess the associations between methionine intake (total, plant, and animal sources) and cognitive function among adults. Furthermore, we explored the interaction between methionine (total, plant, and animal sources) and lead intake in relation to cognitive function.

## 2. Materials and Methods

### 2.1. The Study Design and Study Sample

The present study was a longitudinal study based on repeated measurements of data on dietary intake and cognitive function in the CHNS, which is an ongoing open-cohort international collaborative project between the Carolina Population Center at the University of North Carolina at Chapel Hill and the National Institute for Nutrition and Health (NINH) at the China Centers for Disease Control and Prevention (CCDC). The CHNS applied a multistage, stratified cluster sampling in 11 waves of data collection (1989, 1991, 1993, 1997, 2000, 2004, 2006, 2009, 2011, 2015, and 2018) and with more than 30,000 participants recruited from 15 provinces in China [29]. Cognitive function screening tests were conducted among those aged over 55 years in the 1997, 2000, 2004, and 2006 surveys, so we only used data from between 1997 and 2006. In total, 4852 participants had at least one cognitive test between 1997 and 2006. Of these, 3302 attended the screening test in ≥2 surveys. Our analyses were limited to participants who completed at least one cognitive screening test.

The study’s data collection was approved by the Carolina Population Center at the University of North Carolina at Chapel Hill, as well as by the CCDC. Written informed consent was obtained from all participants before any data were collected.

### 2.2. Outcome Variable: Cognitive Function Assessment

Objective measures for global cognitive function and self-reported measures for memory were used to assess cognitive function.

Global cognitive function: The cognitive screening was conducted through a face-to-face interview and included a subset of items from the Telephone Interview for Cognitive Status–Modified by trained health professionals in the CHNS [30]. The screening included three assignments: (1) immediate recall of 10 words and recall of the same list of words four minutes later (10 points each), (2) counting down from 20 to 1 (2 points each), and (3) subtracting from 100 in intervals of 7 (5 points each). The overall cognitive total score ranged from 0 to 27. Higher cognitive scores represent better cognitive abilities. We chose the first quartile of cognitive function test scores, equivalent to a cutoff of <7 for the overall cognitive function score, to represent poor cognitive function. A study in Shanghai reported that the prevalence of mild cognitive impairment in people aged 60 years and older was 20% [31].

Self-reported memory: Participants were asked about self-reported memory with the question “How is your memory?”: (1) very good, (2) good, (3) ok, (4) bad, (5) very bad, or (9) unknown; participants who answered with “bad” or “very bad” were rated as having poor memory. Changes in memory were assessed by the question “How has your memory changed in the past 12 months?”: (1) improved, (2) stayed the same, (3) declined, or (9) unknown. Participants who reported “declined” were considered to have experienced memory loss.

### 2.3. Exposure Variable: Dietary Intake of Methionine and Lead

Individual dietary intake data were collected on three consecutive days by trained investigators for each wave. In addition, foods and condiments in the home inventory, along with foods purchased from markets or harvested from gardens, were weighed and recorded by interviewers at the beginning and the end of the 3-day survey period. A detailed description of dietary measurements has been published previously [32], and the dietary assessment method has been validated [33]. Using the 3-day average food intake data, the intake of nutrients—including total, animal, and plant methionine and lead—was calculated according to the Chinese Food Composition Table [34]. We calculated the cumulative mean total, animal, and plant methionine and lead intakes for each individual at each period to reduce intraindividual variability and represent long-term habitual intake [35]. For example, if a participant participated in the surveys in 1991, 1993, and 1997, at the age of 55, 57, and 61 years and with an intake of x, y, and z, respectively, the cumulative mean intake was calculated as (x + y + z)/3. Individual dietary lead intake was estimated based on the food intake described above and calculated using published food lead concentration data (i.e., the mean lead concentration in each food category) from Jiangsu Province (one of the nine provinces in the CHNS) [36]. The lead level (μg/d) table was based on lead measurements in 2077 food samples from 23 food categories during 2007–2010.

### 2.4. Covariates

A structured questionnaire was used to collect data on sociodemographic and lifestyle factors. The following constructed variables were included to reflect socioeconomic status (SES): education (low: illiterate/primary school; medium: junior middle school; and high: high middle school or higher), per capita annual family income (recoded into tertiles as low, medium, and high), and urbanization levels (recoded into tertiles as low, medium, and high) [32].

In addition, the following factors were considered as potential confounders in our analysis. Smoking status was categorized into nonsmokers, ex-smokers, and current smokers. Alcohol drinking was categorized as yes or no. Physical activity levels (metabolic equivalents of tasks) were estimated on the basis of self-reported activities (including occupational, domestic, transportation, and leisure-time physical activity) and their duration using a Compendium of Physical Activities [37]. BMI was calculated as weight (kg) divided by height squared (m^2^). Overweight and obesity was defined as BMI ≥ 24.0 kg/m^2^ according to the Guidelines for the Prevention and Control of Overweight and Obesity in Chinese Adults [38]. Diabetes and stroke were self-reported and recorded as “yes” or “no”.

### 2.5. Statistical Analyses

Cumulative mean methionine was recorded in quartiles. Descriptive statistics were calculated using the mean ± standard deviation (SD) for continuous variables or *n* (%) for categorical variables. Baseline characteristics according to the quartiles of total methionine intake were compared using one-way analysis of variance (ANOVA) for continuous variables and chi-squared tests for categorical variables. The association between methionine intake and cognitive function was investigated using mixed-effect regression analysis. A negative regression coefficient indicates a decline in cognitive function. A set of four multivariable models were used: Model 1 was adjusted for age, gender, and energy intake; Model 2 was further adjusted for education, income, urbanization, smoking, alcohol drinking, and physical activity; Model 3 was further adjusted for intake of fruit and vegetables, BMI, hypertension, self-reported diabetes, and stroke; Model 4 was the same as Model 3 but excluded those who only attended one wave of the survey. In the subgroup analyses, the multiplicative interaction among animal- and plant-based methionine intake, lead intake, and covariates (i.e., sex, age, education, income, urbanization, smoking, overweight, and hypertension) was summed by adding a product term to the regression model. We applied a cutoff point of 60 years for age subgroup analysis due to the fact that the general retirement age in China is 60. The interaction between methionine and lead intakes was visualized using the marginplot function in Stata.

All statistical analyses were conducted using Stata 17.0 (Stata Corporation, College Station, TX, USA). Significance was considered when *p* < 0.05 (2-sided).

## 3. Results

### 3.1. Descriptive Results

Table 1 illustrates the sample characteristics of 4661 participants who attended the first cognitive function test based on the quartiles of total methionine intake. The cumulative mean ± SD methionine intake was 1910.5 ± 334.0 mg/d (animal, 916.6 ± 454.1; plant, 993.9 ± 345.1) in the highest quartile (Q4). Across the quartiles of methionine intake, the intakes of energy, protein, fruit, fresh vegetables, meat, and lead were increased, while the prevalence of poor cognitive function (global cognition score < 7) decreased from 27.8% to 10.8%. Higher methionine intakes were associated with higher BMI. The prevalence of self-reported poor memory and memory decline also decreased with the increase in methionine intake. Across quartiles of animal methionine intake, the intake of lead was slightly different (lowest in Q2 and highest in Q4, 96.8 µg/d vs. 109.5 µg/d) (Supplement Table S1a). Lead intake increased from 79.8 µg/d in Q1 to 124.2 µg/d in Q4 of plant methionine intake (Supplementary Table S1b).

### 3.2. Associations between Total, Animal, and Plant Methionine Intake and Cognition

Mixed-effect models showed the associations between total, animal-based, and plant-based methionine intakes and cognition in adults aged 55 years (Table 2). In Model 3, after adjusting for sociodemographic and lifestyle factors and health conditions, the regression coefficients (95% CIs) for global cognition score across Q1-4 of total methionine intake were 0.00, 0.08 (−0.31, 0.47), 0.34 (−0.08, 0.76), and 0.54 (0.08, 1.01), respectively. When stratified by protein source, a similar positive association between animal methionine intake and cognitive function was observed in all models, and the regression coefficients (95% CIs) in the fully adjusted models were 0.00, 0.63 (0.19, 1.06), 1.15 (0.67, 1.62), and 1.80 (1.27, 2.32), respectively. In contrast, higher plant methionine intake was more likely to show a lower cognitive score, and the regression coefficients (95% CIs) were 0.00, −0.86 (−1.28, −0.44), −0.98 (−1.45, −0.52), and −1.79 (−2.33, −1.24) across the quartiles of intake, respectively. Similar associations were found for total methionine intake, animal methionine intake, and plant methionine intake with global cognition score < 7, self-reported poor memory, and self-reported memory decline (Supplementary Table S2).

### 3.3. Lead Intake Status Modifies the Association between Animal Methionine Intake and Cognitive Function

**Figure 1:**
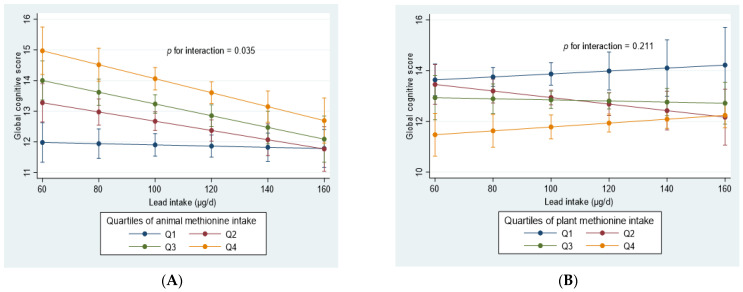
Interaction of animal (**A**) and plant methionine (**B**) intake with lead intake in relation to cognition among adults participating in the China Health and Nutrition Survey (CHNS) during 1997–2006. Values are means (95% CIs) derived by using the margins command in Stata after running a mixed linear model adjusted for age, gender, energy intake, education, income, urbanization, smoking, alcohol drinking, physical activity, intake of fruit and vegetables, BMI, hypertension, self-reported diabetes, and stroke. All participants who attended at least two waves of the survey were included in the analyses. Q = quartile.

Interestingly, we identified a significant interaction (*p* = 0.035) between animal methionine intake and lead intake with respect to global cognitive function scores (Figure 1A). The positive association between animal methionine intake and cognitive function was stronger among those with lower lead intake than in those with higher lead intake. However, no significant interaction (*p* = 0.211) existed between plant methionine intake and lead intake (Figure 1B).

### 3.4. Subgroup Analyses of the Associations between Quartiles of Animal or Plant Methionine Intakes and Global Cognition Scores

Similarly, there were interactions between age (*p* = 0.002), education (*p* = 0.007), income (*p* = 0.016), urbanization (*p* = 0.045), and animal methionine intake in relation to global cognition scores (Table 3). The positive associations were stronger in those with higher levels of urbanization and older age. Across the quartiles of animal methionine intake, the regression coefficients (95% CIs) were 0.00, 0.60 (0.12 to 1.08), 1.46 (0.94 to 1.98), and 2.13 (1.54 to 2.71) in individuals aged ≥ 60, respectively, while they were 0.00, 0.99 (0.15 to 1.82), 1.13 (0.32 to 1.95), and 1.76 (0.92 to 2.59) in those who lived in high levels of urbanization, respectively.

In contrast to the positive relationship between animal methionine intake and cognitive function, the negative associations with plant methionine intake were stronger in higher levels of urbanization (Table 4). Across the quartiles of plant methionine intake, the regression coefficients (95% CIs) were 0.00, −0.62 (−1.10 to −0.15), −0.85 (−1.41 to −0.28), and −2.14 (−2.91 to −1.37) in high level of urbanization, respectively. There was no interaction with age, education, income, smoking, hypertension status, or overweight and obesity.

## 4. Discussion

This longitudinal study of older adults living in China identified that methionine intakes from animal and plant sources had different effects on cognition. Higher total and animal methionine intakes were more likely to have a positive impact on cognitive function, while higher plant methionine intake was associated with a higher risk of impaired cognition. In addition, animal methionine intake had remarkable interactions with lead intake, but this was not the case for plant methionine; the positive association between animal methionine intake and cognition was much stronger in those with lower lead intake than those with higher levels. To the best of our knowledge, this is the first longitudinal study to report such associations in older adults.

The functional relevance of methionine intake for cognition in the general population remains to be elucidated. In vitro, some studies have indicated that methionine restriction had a beneficial effect on cognition [11,12], while others reported that methionine supplementation could improve cognition in rat models [13,26,27]. To the best of our knowledge, although a few studies have shown protective effects of methionine on cognition in human tissues [15,16], there is no supportive evidence to clarify this critical association in adults using longitudinal data. Furthermore, the same nutrients from different sources may show different effects on health [39]. Herein, we found that higher animal methionine intake was positively associated with cognition, but higher plant methionine intake had the opposite effect on cognitive function.

To date, various studies have reported positive associations between dietary protein intake and cognition [40]. Animal-derived proteins contain adequate proportions of all essential amino acids, making them complete proteins. On the other hand, the vast majority of plant proteins are incomplete proteins, except for some—such as soybeans. Consistently, previous studies conducted in older adults have reported that compared with the lowest quartile (Q1), those higher protein intake (Q3 or Q4) from animal sources were three times more likely to have better cognitive performance, whereas plant protein intake had no association with cognition [39,41]. In the present study, the animal methionine intake increased by 3.5-fold from Q1 to Q4 of total methionine intake, accompanied by higher intake of protein and meat, but the absolute increase was relatively small in terms of plant methionine intake. Thus, we propose that the beneficial effects of animal methionine may partially be attributed to higher optimal protein intake, which can retain or help improve cognition.

Since the opening and reform at the end of the 1970s, rapid industrialization and urbanization have further aggravated soil pollution in China. According to the report issued by the Ministry of Environmental Protection and the Ministry of Land Resources, about 19.4% of the arable land of the country is polluted [42]. Lead, as one of the key heavy metals, is difficult to excrete, and its accumulation leads to serious cognitive decline. Prior evidence has indicated that methionine supplementation in lead-exposed rats led to improved cognitive function via the NMDAR NR1 mRNA and protein expression [26,27]. Bilen and colleagues also reported that methionine can decrease the expression of cortical NMDAR subunits through an epigenetic mechanism including increased histone methyltransferase and inhibit the levels of histone H3 lysine (K9) trimethylation, resulting in beneficial effects for attenuating stress in a murine model [14]. Despite methionine metabolism playing an important role in the regulation of the NMDAR pathway, there exist enormous gaps in the knowledge of their interaction in relation to cognition, which warrant further investigations. On the other hand, it has also been reported that methionine treatment increased the fecal excretion of lead and promoted the restoration of the lead-induced decrease in hepatic glutathione levels [28]. This evidence suggests an interactive effect between dietary methionine and lead intake on cognitive function. Accordingly, we found that the beneficial effect of animal methionine on cognition was only found in those who had lower lead intake, and the protective effect disappeared when lead intake was high. Our results provide novel evidence for the effects of nutrient–nutrient interactions on cognition.

The positive association of animal methionine intake with cognition was much stronger in individuals aged ≥60 years than in their younger counterparts. This disparity may be due to the higher prevalence of impaired cognition in older adults, and this may enhance the effects of increased animal protein intake on cognition. A meta-analysis of 53 studies with 123,766 Chinese subjects published in 2021 reported that the prevalence of impaired cognition increased from 11.1% in adults aged 60–69 years to 38.0% in those aged over 90 years [43]. Additionally, a positive association between animal methionine intake and cognition was identified in the low-education group, while this association became negative in the high-education group. In general, an individual with a higher education is more likely to have better nutritional intake and meet nutritional recommendations. However, this association is inconsistent. Some early studies reported that higher educational attainment was correlated with cognitive decline [44,45], but this was not the case in later publications [46,47].

The inverse association between plant methionine intake and cognition was unexpected. Methionine is most abundant in red meat, while it is scarce in a vegan diet [48]. This inverse association could be due to the fact that a high ratio of plant-based food in the Chinese food culture represents a relatively low nutritional quality (e.g., low in meat intake), in addition to high intake of iron and heavy metals such as lead [36,49]. In the present study, in the highest quartile of plant methionine intake, the mean intake of meat was much lower (41.5 vs. 144.7 g/d) than for those in the highest quartile of animal methionine intake. The difference in lead intake was much greater across the quartiles of plant methionine intake than those of animal methionine intake. Furthermore, the inverse association was stronger in those living in more urbanized areas, which are closely associated with higher heavy mental contamination [21]. A recent meta-analysis of 13 population studies suggested that vegans have a higher risk of mental problems compared to omnivores, which may be due to the protective effects of the intake of several amino acids by omnivores—such as methionine—against depression and anxiety [50]. On the other hand, higher urbanization was more likely to be associated with an optimal animal protein food supply; thus, a slightly stronger association was observed between animal methionine intake and global cognition scores. Finally, unlike the significant interaction of income and education levels with animal methionine intake in relation to cognition, we failed to observe their interaction with plant methionine intake. Despite a rapid nutritional transition accompanied by socioeconomic development, the CHNS data showed that plant-based food was still the main component of Chinese diets [51], which may weaken the influence of income and education on the association between plant methionine intake and cognition. More studies are needed to elucidate the association between plant methionine intake and cognition.

The prevalence of obesity in China has been increasing since the 1990s and is now a major public health problem, with one in every two adults being overweight or obese [52]. The association between obesity and cognitive health is receiving increasing recognition. It was suggested that obesity-associated systemic inflammation leads to inflammation within the brain, and that this is partially responsible for impaired cognition [53]. Two longitudinal studies with a more than 27-year follow-up consistently reported that adults with obesity (≥30 kg/m^2^) at mid-life (about 40–45 years) had a greater risk of developing dementia in later life [54,55], whereas in the present study we found that the association between methionine intake was independent of BMI. This result may suggest that mechanisms other than obesity are responsible for the association between methionine intake and cognition.

The major strength of our study was that we used the CHNS database—a prospective, large-sample-size cohort—to make repeated measurements of dietary intake and cognitive function. The data were obtained through a 24 h diet record recall for 3 consecutive days, increasing the reliability and validity of long-term methionine intake. Due to the large sample size from different cities and rural areas in different provinces in the country, our results are highly generalizable. Additionally, the association between lead and cognitive function could be analyzed because there was a large gap in lead intake among the study population. Lastly, we controlled for different known and potential confounding factors in our analyses. The present study expanded on previous findings and first supported the positive link between methionine intake—particularly animal methionine intake—and cognitive function in humans.

Despite its strengths and implications for future research, our study had limitations. Because of the influence of different cognitive states, it was difficult to avoid memory bias during the 24 h food recall. Although we controlled for many factors to reduce confounding, we could not exclude the possibility of residual confounding. Secondly, lead intake was mainly estimated based on food lead concentration data from Jiangsu Province; because regional differences are likely to be large, more studies are warranted for verification. Thirdly, the study did not have information on traumatic brain injury, thyroid function, and chronic diseases such as COPD. It has been shown that these conditions are associated with cognition [7,8,9]. Finally, we were not able to explore potential mechanisms due to a lack of methionine-related or lead-related biomarkers.

In conclusion, the present study found that higher total and animal methionine intakes are associated with better cognitive function among Chinese older adults, independent of lifestyle and sociodemographic factors. There was a significant interaction between animal methionine intake and lead intake, showing improved cognitive function in those with higher methionine intake and lower lead intake. Further research is needed to elucidate the relationship between methionine intake and lead intake in relation to cognitive function. 

## Figures and Tables

**Table 1:** Sample characteristics of Chinese adults aged ≥55 years attending the first cognitive function test, by quartiles of cumulative total methionine intake (*n* = 4661).

	Q1	Q2	Q3	Q4	*p*
	*n* = 1166	*n* = 1165	*n* = 1165	*n* = 1165	
Age (years)	66.8 ± 9.0	63.5 ± 7.6	62.1 ± 6.9	61.3 ± 6.3	<0.001
Survey year					<0.001
1997	585 (50.2%)	557 (47.8%)	487 (41.8%)	423 (36.3%)	
2000	187 (16.0%)	217 (18.6%)	202 (17.3%)	191 (16.4%)	
2004	260 (22.3%)	251 (21.5%)	278 (23.9%)	317 (27.2%)	
2006	134 (11.5%)	140 (12.0%)	198 (17.0%)	234 (20.1%)	
Sex					<0.001
Men	374 (32.1%)	460 (39.5%)	615 (52.8%)	788 (67.6%)	
Women	792 (67.9%)	705 (60.5%)	550 (47.2%)	377 (32.4%)	
**Socioeconomic factors**					
Income					<0.001
Low	467 (40.6%)	405 (35.0%)	347 (30.0%)	244 (21.3%)	
Medium	365 (31.7%)	400 (34.6%)	347 (30.0%)	284 (24.8%)	
High	319 (27.7%)	351 (30.4%)	464 (40.1%)	618 (53.9%)	
Education					<0.001
Low	831 (84.2%)	854 (81.0%)	752 (69.6%)	611 (56.3%)	
Medium	85 (8.6%)	119 (11.3%)	192 (17.8%)	227 (20.9%)	
High	71 (7.2%)	81 (7.7%)	137 (12.7%)	248 (22.8%)	
Urbanization					<0.001
Low	359 (30.8%)	352 (30.2%)	277 (23.8%)	195 (16.7%)	
Medium	345 (29.6%)	377 (32.4%)	293 (25.2%)	283 (24.3%)	
High	462 (39.6%)	436 (37.4%)	595 (51.1%)	687 (59.0%)	
**Lifestyle factor**					
Smoking					<0.001
Nonsmokers	864 (74.5%)	827 (71.1%)	759 (65.3%)	682 (58.5%)	
Ex-smokers	43 (3.7%)	36 (3.1%)	35 (3.0%)	56 (4.8%)	
Current smokers	252 (21.7%)	300 (25.8%)	369 (31.7%)	427 (36.7%)	
Alcohol drinking	267 (23.4%)	293 (25.7%)	401 (35.3%)	471 (40.9%)	<0.001
Physical activity (MET)	72.7 ± 94.0	95.1 ± 104.5	93.7 ± 99.8	89.5 ± 96.8	<0.001
**Weight status**					
BMI (kg/m^2^)	22.4 ± 3.8	22.9 ± 3.7	23.4 ± 3.6	23.5 ± 3.3	<0.001
Overweight and obesity (BMI ≥ 24 kg/m^2^)	326 (30.9%)	374 (34.5%)	450 (40.9%)	457 (41.9%)	<0.001
**Dietary intakes**					
Energy intake (kcal/d)	1661.6 ± 458.8	1998.7 ± 516.0	2227.0 ± 548.7	2486.1 ± 660.7	<0.001
Fat intake (g/d)	49.4 ± 29.2	60.0 ± 32.8	70.3 ± 34.2	87.1 ± 39.0	<0.001
Protein intake (g/d)	45.2 ± 12.6	58.1 ± 15.3	67.9 ± 17.7	83.2 ± 25.4	<0.001
Carbohydrate intake (g/d)	256.4 ± 79.7	302.0 ± 97.7	323.4 ± 107.2	332.2 ± 119.6	<0.001
Cumulative methionine intake (mg/d)	915.7 ± 153.9	1219.8 ± 66.4	1450.1 ± 73.4	1910.5 ± 334.0	<0.001
Cumulative animal methionine intake (mg/d)	203.6 ± 161.9	348.7 ± 211.3	517.3 ± 263.7	916.6 ± 454.1	<0.001
Cumulative plant methionine intake (mg/d)	712.0 ± 186.0	871.1 ± 208.3	932.8 ± 258.3	993.9 ± 345.1	<0.001
Most recent methionine intake(mg/d)	886.1 ± 237.8	1173.3 ± 267.4	1429.8 ± 346.6	1914.5 ± 669.8	<0.001
Most recent animal methionine intake (mg/d)	225.5 ± 211.6	382.2 ± 284.2	564.9 ± 364.1	982.2 ± 689.2	<0.001
Most recent plant methionine intake (mg/d)	660.7 ± 212.6	791.1 ± 243.8	865.0 ± 306.4	932.4 ± 372.7	<0.001
Lead intake (µg/d)	80.8 ± 26.7	97.5 ± 27.6	107.0 ± 31.6	121.6 ± 36.5	<0.001
Intake of fruit (g/d)	13.4 ± 50.1	17.5 ± 70.8	23.1 ± 76.6	39.0 ± 107.9	<0.001
Intake of fresh vegetables (g/d)	224.4 ± 152.9	265.4 ± 159.4	287.9 ± 180.5	322.3 ± 196.9	<0.001
Intake of meat (g/d)	32.9 ± 43.1	55.4 ± 57.0	81.5 ± 75.5	127.3 ± 106.7	<0.001
**Disease history**					
Hypertension	424 (39.0%)	375 (34.1%)	373 (33.4%)	394 (35.5%)	0.028
Diabetes	34 (3.0%)	34 (3.0%)	34 (3.0%)	47 (4.1%)	0.31
Stroke	34 (3.0%)	18 (1.6%)	24 (2.1%)	24 (2.1%)	0.16
**Cognitive function**					
Self-reported poor memory	350 (30.3%)	243 (21.1%)	220 (19.0%)	151 (13.1%)	<0.001
Self-reported memory decline	561 (49.5%)	474 (41.9%)	402 (35.5%)	342 (30.2%)	<0.001
Global cognition score	11.3 ± 6.9	12.7 ± 6.5	14.0 ± 6.6	14.9 ± 6.4	<0.001
Global cognition score < 7	324 (27.8%)	221 (19.0%)	166 (14.2%)	126 (10.8%)	<0.001

Data are presented as the mean ± SD for continuous measures and as *n* (%) for categorical measures.

**Table 2:** Association between total, animal, and plant methionine intake and global cognition scores among adults participating in the CHNS.

	Q1	Q2	Q3	Q4	*p* _trend_
Total methionine					
Model 1	0.00	0.48 (0.12 to 0.83)	1.16 (0.78 to 1.55)	1.88 (1.47 to 2.30)	<0.001
Model 2	0.00	0.17 (−0.20 to 0.55)	0.47 (0.07 to 0.88)	0.57 (0.13 to 1.02)	0.008
Model 3	0.00	0.08 (−0.31 to 0.47)	0.34 (−0.08 to 0.76)	0.54 (0.08 to 1.01)	0.013
Model 4	0.00	−0.08 (−0.50 to 0.34)	0.20 (−0.25 to 0.65)	0.37 (−0.13 to 0.87)	0.103
Animal methionine					
Model 1	0.00	1.13 (0.77 to 1.48)	2.40 (2.04 to 2.76)	3.53 (3.16 to 3.90)	<0.001
Model 2	0.00	0.62 (0.23 to 1.01)	1.34 (0.92 to 1.77)	1.88 (1.41 to 2.36)	<0.001
Model 3	0.00	0.57 (0.17 to 0.98)	1.18 (0.73 to 1.62)	1.80 (1.31 to 2.29)	<0.001
Model 4	0.00	0.63 (0.19 to 1.06)	1.15 (0.67 to 1.62)	1.80 (1.27 to 2.32)	<0.001
Plant methionine					
Model 1	0.00	−0.97 (−1.31 to −0.62)	−1.77 (−2.14 to −1.39)	−3.34 (−3.75 to −2.93)	<0.001
Model 2	0.00	−0.61 (−0.99 to −0.23)	−0.78 (−1.20 to −0.37)	−1.69 (−2.18 to −1.21)	<0.001
Model 3	0.00	−0.73 (−1.12 to −0.34)	−0.83 (−1.26 to −0.41)	−1.72 (−2.22 to −1.22)	<0.001
Model 4	0.00	−0.86 (−1.28 to −0.44)	−0.98 (−1.45 to −0.52)	−1.79 (−2.33 to −1.24)	<0.001

Values are regression coefficients and 95% CIs from mixed-effect linear models. Q = quartile. Model 1 was adjusted for age, gender, and energy intake. Model 2 was further adjusted for education, income, urbanization, smoking, alcohol drinking, and physical activity. Model 3 was further adjusted for intake of fruit and vegetables, BMI, hypertension, self-reported diabetes, and stroke. Model 4 was the same as Model 3 but excluded those who attended only one wave of the survey.

**Table 3:** Subgroup analyses of the association between quartiles of animal methionine intake and global cognition scores.

	Q1	Q2	Q3	Q4	*p* _trend_	*p* _interaction_
Age (years)						0.002
<60	0.00	0.61 (−0.12 to 1.34)	0.69 (−0.09 to 1.47)	1.10 (0.25 to 1.96)	0.017	
≥60	0.00	0.60 (0.12 to 1.08)	1.46 (0.94 to 1.98)	2.13 (1.54 to 2.71)	<0.001	
Sex						0.050
Men	0.00	0.66 (0.03 to 1.28)	1.39 (0.73 to 2.06)	1.74 (1.03 to 2.46)	<0.001	
Women	0.00	0.48 (−0.05 to 1.00)	0.96 (0.38 to 1.55)	1.87 (1.20 to 2.54)	<0.001	
Education						0.007
Low	0.00	0.63 (0.19 to 1.08)	1.48 (0.98 to 1.98)	2.15 (1.57 to 2.72)	<0.001	
Medium	0.00	0.09 (−1.14 to 1.31)	−0.43 (−1.67 to 0.80)	0.99 (−0.31 to 2.30)	0.072	
High	0.00	−2.21 (−4.18 to −0.23)	−1.70 (−3.60 to 0.21)	−1.91 (−3.80 to −0.02)	0.395	
Income						0.016
Low	0.00	0.28 (−0.32 to 0.88)	1.27 (0.53 to 2.01)	2.41 (1.52 to 3.30)	<0.001	
Medium	0.00	0.42 (−0.25 to 1.10)	1.11 (0.39 to 1.83)	1.73 (0.89 to 2.56)	<0.001	
High	0.00	1.21 (0.33 to 2.10)	1.00 (0.13 to 1.87)	1.52 (0.62 to 2.42)	0.006	
Urbanization						0.045
Low	0.00	0.73 (0.05 to 1.40)	1.03 (0.17 to 1.88)	1.37 (0.12 to 2.62)	0.003	
Medium	0.00	0.09 (−0.57 to 0.75)	1.38 (0.63 to 2.12)	2.08 (1.21 to 2.95)	<0.001	
High	0.00	0.99 (0.15 to 1.82)	1.13 (0.32 to 1.95)	1.76 (0.92 to 2.59)	<0.001	
Overweight/obesity						0.362
No	0.00	0.41 (−0.04 to 0.86)	1.09 (0.59 to 1.59)	1.71 (1.14 to 2.27)	<0.001	
Yes	0.00	1.15 (0.27 to 2.04)	1.46 (0.54 to 2.38)	2.05 (1.07 to 3.04)	<0.001	

Values are means (95% CIs) derived by using the margins command in Stata after running a mixed linear model adjusted for age, gender, energy intake, education, income, urbanization, smoking, alcohol drinking, physical activity, intake of fruit and vegetables, BMI, hypertension, self-reported diabetes, and stroke. All participants who attended at least two waves of the survey were included in the analyses. Q = quartile. Stratification variables were not adjusted in the corresponding models.

**Table 4:** Subgroup analyses of the associations between quartiles of plant methionine intake and global cognition scores.

	Q1	Q2	Q3	Q4	*p* _trend_	*p* _interaction_
Age (years)						0.497
<60	0.00	−0.71 (−1.52 to 0.09)	−0.61 (−1.43 to 0.22)	−1.87 (−2.79 to −0.94)	<0.001	
≥60	0.00	−0.76 (−1.20 to −0.31)	−0.99 (−1.49 to −0.49)	−1.65 (−2.25 to −1.05)	<0.001	
Sex						0.034
Men	0.00	−0.53 (−1.19 to 0.14)	−1.20 (−1.88 to −0.51)	−1.78 (−2.55 to −1.02)	<0.001	
Women	0.00	−0.82 (−1.30 to −0.33)	−0.38 (−0.94 to 0.19)	−1.71 (−2.40 to −1.02)	<0.001	
Education						0.530
Low	0.00	−0.67 (−1.15 to −0.19)	−0.78 (−1.30 to −0.26)	−1.71 (−2.32 to −1.11)	<0.001	
Medium	0.00	−1.60 (−2.54 to −0.66)	−1.61 (−2.64 to −0.59)	−2.65 (−3.88 to −1.42)	<0.001	
High	0.00	−0.16 (−1.04 to 0.71)	−0.37 (−1.40 to 0.67)	−0.79 (−2.18 to 0.60)	0.274	
Income						0.309
Low	0.00	−0.75 (−1.53 to 0.03)	−0.91 (−1.71 to −0.10)	−1.61 (−2.50 to −0.71)	<0.001	
Medium	0.00	−0.12 (−0.84 to 0.59)	−0.40 (−1.18 to 0.37)	−1.49 (−2.37 to −0.60)	<0.001	
High	0.00	−1.13 (−1.68 to −0.57)	−1.04 (−1.68 to −0.40)	−1.93 (−2.75 to −1.12)	<0.001	
Urbanization						0.007
Low	0.00	0.75 (−0.36 to 1.86)	0.47 (−0.60 to 1.55)	−0.65 (−1.77 to 0.46)	0.017	
Medium	0.00	−1.53 (−2.41 to −0.65)	−1.41 (−2.31 to −0.51)	−1.92 (−2.90 to −0.93)	0.002	
High	0.00	−0.62 (−1.10 to −0.15)	−0.85 (−1.41 to −0.28)	−2.14 (−2.91 to −1.37)	<0.001	
Overweight/obesity						0.539
No	0.00	−0.65 (−1.11 to −0.18)	−0.74 (−1.24 to −0.23)	−1.62 (−2.20 to −1.03)	<0.001	
Yes	0.00	−0.89 (−1.59 to −0.18)	−1.09 (−1.87 to −0.31)	−2.11 (−3.07 to −1.14)	<0.001	

Values are means (95% CIs) derived by using the margins command in Stata after running a mixed linear model adjusted for age, gender, energy intake, education, income, urbanization, smoking, alcohol drinking, physical activity, intake of fruit and vegetables, BMI, hypertension, self-reported diabetes, and stroke. All participants who attended at least two waves of the survey were included in the analyses. Q = quartile. Stratification variables were not adjusted in the corresponding models.

## Data Availability

The datasets generated and analyzed during the present study are available in the CHNS repository at https://www.cpc.unc.edu/projects/china.

## Supplementary Materials

The following supporting information can be downloaded at: https://www.mdpi.com/article/10.3390/nu14214561/s1, Table S1: (a) Sample characteristics of Chinese adults aged ≥55 years old attending the first cognitive function test by quartiles of cumulative animal methionine intake (n = 4661); (b) Sample characteristics of Chinese adults aged ≥55 years old attending the first cognitive function test by quartiles of cumulative plant methionine intake (n = 4661); Table S2: Association between quartiles of methionine intake and cognition among Chinese adults.

## References

[1] WHO (2022). Dementia. https://www.who.int/en/news-room/fact-sheets/detail/dementia.

[2] Jia L., Du Y., Chu L., Zhang Z., Li F., Lyu D., Li Y., Li Y., Zhu M., Jiao H. (2020). Prevalence, risk factors, and management of dementia and mild cognitive impairment in adults aged 60 years or older in China: A cross-sectional study. Lancet Public Health.

[3] National Bureau of Statistics The Seventh National Census of China. http://www.stats.gov.cn/tjsj/ndsj/2021/indexch.htm.

[4] Zhang Q.L., Wu Y.L., Han T.K., Liu E.P. (2019). Changes in Cognitive Function and Risk Factors for Cognitive Impairment of the Elderly in China: 2005–2014. Int. J. Environ. Res. Public Health.

[5] Han F., Luo C., Lv D., Tian L., Qu C. (2022). Risk Factors Affecting Cognitive Impairment of the Elderly Aged 65 and Over: A Cross-Sectional Study. Front. Aging Neurosci..

[6] Senee A., Ishnoo Y.B., Jeewon R. (2022). An Analysis of the Contributors and Factors Influencing Dietary Patterns among the Elderly Population. Curr. Res. Nutr. Food Sci. J..

[7] Giannouli V., Toulis K.A., Syrmos N. (2014). Cognitive function in Hashimoto’s thyroiditis under levothyroxine treatment. Hormones.

[8] Dodd J.W., Getov S.V., Jones P.W. (2010). Cognitive function in COPD. Eur. Respir. J..

[9] Rabinowitz A.R., Levin H.S. (2014). Cognitive sequelae of traumatic brain injury. Psychiatr. Clin..

[10] Martínez Y., Li X., Liu G., Bin P., Yan W., Más D., Valdivie M., Hu C.A.A., Ren W.K., Yin Y. (2017). The role of methionine on metabolism, oxidative stress, and diseases. Amino Acids.

[11] Ren B., Wang L., Shi L., Jin X., Liu Y., Liu R.H., Yin F., Cadenas E., Dai X., Liu Z. (2021). Methionine restriction alleviates age-associated cognitive decline via fibroblast growth factor 21. Redox Biol..

[12] Jove M., Ayala V., Ramirez-Nunez O., Naudi A., Cabre R., Spickett C.M., Portero-Otín M., Pamplona R. (2013). Specific lipidome signatures in central nervous system from methionine-restricted mice. J. Proteome Res..

[13] Troen A.M., Chao W.H., Crivello N.A., D’Anci K.E., Shukitt-Hale B., Smith D.E., Selhub J., Rosenberg I.H. (2008). Cognitive impairment in folate-deficient rats corresponds to depleted brain phosphatidylcholine and is prevented by dietary methionine without lowering plasma homocysteine. J. Nutr..

[14] Bilen M., Ibrahim P., Barmo N., Abou H.E., Karnib N., El H.L., Khalifeh M., Jabre V., Houbeika R., Stephan J.S. (2020). Methionine mediates resilience to chronic social defeat stress by epigenetic regulation of NMDA receptor subunit expression. Psychopharmacology.

[15] Miller A.L. (2003). The methionine-homocysteine cycle and its effects on cognitive diseases. Altern. Med. Rev..

[16] Gabbita S.P., Aksenov M.Y., Lovell M.A., Markesbery W.R. (1999). Decrease in peptide methionine sulfoxide reductase in Alzheimer’s disease brain. J. Neurochem..

[17] Fu J., Liu Q., Zhang M., Sun C., Du Y., Zhu Y., Lin H.Y., Jin M.D., Ma F., Li W. (2022). Association between methionine cycle metabolite-related diets and mild cognitive impairment in older Chinese adults: A population-based observational study. Nutr. Neurosci..

[18] Dong Z., Gao X., Chinchilli V.M., Sinha R., Muscat J., Winkels R., Richie J.J. (2022). Association of dietary sulfur amino acid intake with mortality from diabetes and other causes. Eur. J. Nutr..

[19] Elango R. (2020). Methionine Nutrition and Metabolism: Insights from Animal Studies to Inform Human Nutrition. J. Nutr..

[20] Mason L.H., Harp J.P., Han D.Y. (2014). Pb neurotoxicity: Neuropsychological effects of lead toxicity. Biomed. Res. Int..

[21] Liu X., Song Q., Tang Y., Li W., Xu J., Wu J., Wang F., Brookes P.C. (2013). Human health risk assessment of heavy metals in soil-vegetable system: A multi-medium analysis. Sci. Total Environ..

[22] Reuben A. (2018). Childhood Lead Exposure and Adult Neurodegenerative Disease. J. Alzheimers Dis..

[23] Reuben A., Caspi A., Belsky D.W., Broadbent J., Harrington H., Sugden K., Houts R.M., Ramrakha S., Poulton R., Moffitt T.E. (2017). Association of Childhood Blood Lead Levels With Cognitive Function and Socioeconomic Status at Age 38 Years and With IQ Change and Socioeconomic Mobility Between Childhood and Adulthood. JAMA-J. Am. Med. Assoc..

[24] Power M.C., Korrick S., Tchetgen E.J.T., Nie L.H., Grodstein F., Hu H., Weuve J., Schwartz J., Weisskopf M.G. (2014). Lead exposure and rate of change in cognitive function in older women. Environ. Res..

[25] Neal A.P., Guilarte T.R. (2010). Molecular neurobiology of lead (Pb(2+)): Effects on synaptic function. Mol. Neurobiol..

[26] Fan G., Feng C., Wu F., Ye W., Lin F., Wang C., Yan J., Zhu G.C., Xiao Y.M., Bi Y. (2010). Methionine choline reverses lead-induced cognitive and N-methyl-d-aspartate receptor subunit 1 deficits. Toxicology.

[27] Fan G., Feng C., Li Y., Wang C., Yan J., Li W., Feng J.G., Shi X.L., Bi Y. (2009). Selection of nutrients for prevention or amelioration of lead-induced learning and memory impairment in rats. Ann. Occup. Hyg..

[28] Tandon S.K., Singh S., Flora S.J. (1994). Influence of methionine and zinc supplementation during chelation of lead in rats. J. Trace Elem. Electrolytes Health Dis..

[29] Popkin B.M., Du S.F., Zhai F.Y., Zhang B. (2010). Cohort Profile: The China Health and Nutrition Survey-monitoring and understanding socio-economic and health change in China, 1989–2011. Int. J. Epidemiol..

[30] Plassman B.L., Welsh K.A., Helms M., Brandt J., Page W.F., Breitner J.C.S. (1995). Intelligence and Education as Predictors of Cognitive State in Late-Life—A 50-Year Follow-Up. Neurology.

[31] Ding D., Zhao Q.H., Guo Q.H., Meng H.J., Wang B., Luo J.F., Mortimer J.A., Borenstein A.R., Hong Z. (2015). Prevalence of mild cognitive impairment in an urban community in China: A cross-sectional analysis of the Shanghai Aging Study. Alzheimers Dement..

[32] Zhai F.Y., Du S.F., Wang Z.H., Zhang J.G., Du W.W., Popkin B.M. (2014). Dynamics of the Chinese diet and the role of urbanicity, 1991–2011. Obes. Rev..

[33] Yao M., McCrory M.A., Ma G., Tucker K.L., Gao S.J., Fuss P., Roberts S.B. (2003). Relative influence of diet and physical activity on body composition in urban Chinese adults. Am. J. Clin. Nutr..

[34] Yang Y. (2005). Chinese Food Composition Table 2004.

[35] Hu F.B., Willett W.C. (2000). Dietary fat and coronary heart disease: A comparison of approaches for adjusting for total energy intake and modeling repeated dietary measurements—Reply. Am. J. Epidemiol..

[36] Jin Y., Liu P., Sun J.F., Wang C.N., Min J., Zhang Y., Wang S.Y., Wu Y.N. (2014). Dietary exposure and risk assessment to lead of the population of Jiangsu province, China. Food Addit. Contam. A.

[37] Ainsworth B.E., Haskell W.L., Whitt M.C., Irwin M.L., Swartz A.M., Strath S.J., O’Brien W.L., Bassett D.R., Schmitz K.H., Emplaincourt P.O. (2000). Compendium of Physical Activities: An update of activity codes and MET intensities. Med. Sci. Sport Exerc..

[38] Zhou B.F. (2002). Predictive values of body mass index and waist circumference for risk factors of certain related diseases in Chinese adults—Study on optimal cut-off points of body mass index and waist circumference in Chinese adults. Biomed. Environ. Sci..

[39] Li Y., Li S., Wang W., Zhang D.F. (2020). Association between Dietary Protein Intake and Cognitive Function in Adults Aged 60 Years and Older. J. Nutr. Health Aging.

[40] Van der Zwaluw N.L., Van de Rest O., Tieland M., Adam J.J., Hiddink G.J., Van Loon L.J.C., De Groot L.C.P.G.M. (2014). The impact of protein supplementation on cognitive performance in frail elderly. Eur. J. Nutr..

[41] Muth A.K., Park S.Q. (2021). The impact of dietary macronutrient intake on cognitive function and the brain. Clin. Nutr..

[42] Chen R., De Sherbinin A., Ye C., Shi G. (2014). China’s soil pollution: Farms on the frontline. Science.

[43] Deng Y., Zhao S.Q., Cheng G.W., Yang J.J., Li B.C., Xu K., Xiao P., Li W.F., Rong S. (2021). The Prevalence of Mild Cognitive Impairment among Chinese People: A Meta-Analysis. Neuroepidemiology.

[44] Albert M.S., Jones K., Savage C.R., Berkman L., Seeman T., Blazer D., Rowe J.W. (1995). Predictors of cognitive change in older persons: MacArthur studies of successful aging. Psychol. Aging.

[45] JacqminGadda H., Fabrigoule C., Commenges D., Dartigues J.F. (1997). A 5-year longitudinal study of the mini-mental state examination in normal aging. Am. J. Epidemiol..

[46] Van Dijk K.R.A., Van Gerven P.W.M., Van Boxtel M.P.J., Van der Elst W., Jolles J. (2008). No protective effects of education during normal cognitive aging: Results from the 6-year follow-up of the Maastricht aging study. Psychol. Aging.

[47] Jansen M.G., Geerligs L., Claassen J.A.H.R., Overdorp E.J., Brazil I.A., Kessels R.P.C., Oosterman J.M. (2021). Positive Effects of Education on Cognitive Functioning Depend on Clinical Status and Neuropathological Severity. Front. Hum. Neurosci..

[48] Schmidt J.A., Rinaldi S., Scalbert A., Ferrari P., Achaintre D., Gunter M.J., Appleby P.N., Key T.J., Travis R.C. (2016). Plasma concentrations and intakes of amino acids in male meat-eaters, fish-eaters, vegetarians and vegans: A cross-sectional analysis in the EPIC-Oxford cohort. Eur. J. Clin. Nutr..

[49] Shi Z., El-Obeid T., Li M., Xu X.Y., Liu J.H. (2019). Iron-related dietary pattern increases the risk of poor cognition. Nutr. J..

[50] Azorin I., Huybrechts I., Moreno L.A., Michels N. (2019). Vegetarianism and veganism versus mental health and cognitive outcomes. A systematic review and meta-analysis. Ann. Nutr. Metab..

[51] Bishwajit G., Ide S., Hossain M.A., Safa M.N. (2014). Trade Liberalization, Urbanization and Nutrition Transition in Asian Countries. J. Nutr. Health Food Sci..

[52] Wang Y., Zhao L., Gao L., Pan A., Xue H. (2021). Health policy and public health implications of obesity in China. Lancet Diabetes Endocrinol..

[53] Miller A.A., Spencer S.J. (2014). Obesity and neuroinflammation: A pathway to cognitive impairment. Brain Behav. Immun..

[54] Xu W.L., Atti A.R., Gatz M., Pedersen N.L., Johansson B., Fratiglioni L. (2011). Midlife overweight and obesity increase late-life dementia risk: A population-based twin study. Neurology.

[55] Whitmer R.A., Gunderson E.P., Barrett-Connor E., Quesenberry C.P., Yaffe K. (2005). Obesity in middle age and future risk of dementia: A 27 year longitudinal population based study. BMJ.

