# Supplementary material for: Interactive Effects of Methionine and Lead Intake on Cognitive Function among Chinese Adults
Source: Nutrients. 2022 Oct 29;14(21):4561. doi: 10.3390/nu14214561 (PMC9656425; doi:10.3390/nu14214561)
Supplement: Supplementary file 1 [file nutrients-14-04561-s001.zip › nutrients-1963476-Supplementary.pdf]

Supplemental Table S1. Sample characteristics of Chinese adults aged ≥55 years old attending the first cognitive function test by quartiles of cumulative methionine intake (n = 4661).

**(a) animal**	Q1 **N=1166**	Q2 **N=1165**	Q3 **N=1165**	Q4 **N=1165**	**p-value**
Age (years)	63.9 (8.2)	63.4 (7.6)	63.6 (8.1)	62.8 (7.1)	0.008
Sex					<0.001
Men	512 (43.9%)	502 (43.1%)	550 (47.2%)	673 (57.8%)	
Women	654 (56.1%)	663 (56.9%)	615 (52.8%)	492 (42.2%)	
Income					<0.001
Low	636 (55.1%)	379 (32.7%)	242 (21.0%)	206 (18.0%)	
Medium	324 (28.1%)	440 (38.0%)	372 (32.3%)	260 (22.7%)	
High	195 (16.9%)	340 (29.3%)	536 (46.6%)	681 (59.4%)	
Education					<0.001
Low	955 (89.8%)	832 (79.2%)	714 (68.9%)	547 (51.8%)	
Medium	79 (7.4%)	134 (12.7%)	185 (17.8%)	225 (21.3%)	
High	29 (2.7%)	85 (8.1%)	138 (13.3%)	285 (27.0%)	
Urbanization					<0.001
Low	657 (56.3%)	294 (25.2%)	158 (13.6%)	74 (6.4%)	
Medium	336 (28.8%)	424 (36.4%)	324 (27.8%)	214 (18.4%)	
High	173 (14.8%)	447 (38.4%)	683 (58.6%)	877 (75.3%)	
Smoking					0.50
Non smoker	769 (66.2%)	796 (68.4%)	789 (68.0%)	778 (66.8%)	
Ex-smokers	48 (4.1%)	39 (3.4%)	33 (2.8%)	50 (4.3%)	
Current smokers	344 (29.6%)	328 (28.2%)	339 (29.2%)	337 (28.9%)	
SURVEY YEAR					<0.001
1997	594 (50.9%)	515 (44.2%)	487 (41.8%)	456 (39.1%)	
2000	189 (16.2%)	192 (16.5%)	217 (18.6%)	199 (17.1%)	
2004	266 (22.8%)	288 (24.7%)	264 (22.7%)	288 (24.7%)	
2006	117 (10.0%)	170 (14.6%)	197 (16.9%)	222 (19.1%)	
Alcohol drinking	324 (28.5%)	344 (30.1%)	355 (31.0%)	409 (35.7%)	0.002
Physical activity (MET)	112.8 (115.7)	94.3 (103.7)	74.1 (87.6)	69.2 (79.4)	<0.001
BMI (kg/m2)	22.1 (3.5)	23.0 (3.7)	23.4 (3.6)	23.7 (3.4)	<0.001
BMI>24 (kg/m2)	276 (26.3%)	388 (36.1%)	444 (40.5%)	499 (45.0%)	<0.001
Energy intake (kcal/d)	2027.0 (655.4)	1986.8 (613.9)	2078.0 (584.0)	2281.4 (619.1)	<0.001
Fat intake (g/d)	45.3 (27.5)	59.0 (31.5)	73.1 (34.0)	89.4 (37.7)	<0.001
Protein intake (g/d)	55.3 (21.4)	57.4 (19.2)	63.6 (20.2)	78.1 (24.0)	<0.001
Carbohydrate intake (g/d)	347.2 (123.3)	301.8 (101.7)	283.6 (91.9)	281.3 (91.3)	<0.001
Cumulative methionine intake (mg/d)	1123.7 (333.0)	1179.2 (257.4)	1375.5 (227.3)	1817.4 (382.9)	<0.001
Cumulative animal methionine intake (mg/d)	78.2 (59.2)	303.3 (71.0)	570.1 (82.4)	1034.7 (329.0)	<0.001
Cumulative plant methionine intake (mg/d)	1045.4 (335.7)	875.9 (250.8)	805.5 (218.7)	782.7 (204.9)	<0.001
Most recent methionine intake(mg/d)	1042.8 (385.0)	1159.5 (388.9)	1365.8 (394.9)	1835.6 (668.2)	<0.001
Most recent animal methionine intake (mg/d)	95.7 (121.2)	356.6 (230.1)	617.1 (277.5)	1085.2 (611.5)	<0.001
Most recent plant methionine intake (mg/d)	947.0 (370.0)	802.8 (291.6)	748.7 (262.1)	750.3 (247.4)	<0.001
Lead intake (ug/d)	102.7 (37.1)	96.8 (34.5)	97.8 (31.1)	109.5 (32.4)	<0.001
Intake of fruit (g/d)	8.1 (60.5)	19.3 (81.6)	25.7 (74.4)	39.8 (95.0)	<0.001
Intake of fresh vegetable (g/d)	274.4 (184.2)	262.0 (179.8)	268.5 (169.7)	295.0 (171.7)	<0.001
Intake of meat (g/d)	12.1 (24.5)	49.8 (48.7)	90.4 (64.2)	144.7 (101.8)	<0.001
Hypertension	356 (33.3%)	390 (35.4%)	395 (35.4%)	425 (37.7%)	0.19
Diabetes	25 (2.2%)	34 (3.0%)	38 (3.3%)	52 (4.6%)	0.013
Stroke	21 (1.8%)	25 (2.2%)	26 (2.3%)	28 (2.4%)	0.78
Self-reported poor memory	335 (29.0%)	249 (21.6%)	214 (18.6%)	166 (14.3%)	<0.001

Self-reported memory decline	546 (48.6%)	456 (40.1%)	420 (37.2%)	357 (31.4%)	<0.001
Global cognition score	11.2 (6.9)	12.8 (6.7)	13.7 (6.4)	15.2 (6.3)	<0.001
Global cognition score < 7	329 (28.2%)	235 (20.2%)	163 (14.0%)	110 (9.4%)	<0.001

(b) plant

	Q1 N=1166	Q2 N=1165	Q3 N=1165	Q4 N=1165	p-value
Age (years)	67.3 (8.8)	63.7 (7.6)	61.7 (6.7)	61.0 (6.1)	<0.001
Sex					<0.001
Men	364 (31.2%)	524 (45.0%)	630 (54.1%)	719 (61.7%)	
Women	802 (68.8%)	641 (55.0%)	535 (45.9%)	446 (38.3%)	
Income					<0.001
Low	289 (25.1%)	307 (26.9%)	356 (30.7%)	511 (44.1%)	
Medium	323 (28.1%)	343 (30.0%)	365 (31.5%)	365 (31.5%)	
High	539 (46.8%)	492 (43.1%)	439 (37.8%)	282 (24.4%)	
Education					<0.001
Low	650 (68.7%)	731 (69.0%)	779 (71.1%)	888 (80.3%)	
Medium	137 (14.5%)	155 (14.6%)	176 (16.1%)	155 (14.0%)	
High	159 (16.8%)	174 (16.4%)	141 (12.9%)	63 (5.7%)	
Urbanization					<0.001
Low	135 (11.6%)	167 (14.3%)	313 (26.9%)	568 (48.8%)	
Medium	216 (18.5%)	319 (27.4%)	392 (33.6%)	371 (31.8%)	
High	815 (69.9%)	679 (58.3%)	460 (39.5%)	226 (19.4%)	
Smoking					<0.001
Non smoker	907 (78.1%)	819 (70.4%)	752 (64.6%)	654 (56.3%)	
Ex-smokers	38 (3.3%)	33 (2.8%)	50 (4.3%)	49 (4.2%)	
Current smokers	216 (18.6%)	311 (26.7%)	362 (31.1%)	459 (39.5%)	
SURVEY YEAR					0.030
1997	535 (45.9%)	536 (46.0%)	494 (42.4%)	487 (41.8%)	
2000	223 (19.1%)	195 (16.7%)	188 (16.1%)	191 (16.4%)	
2004	245 (21.0%)	262 (22.5%)	294 (25.2%)	305 (26.2%)	
2006	163 (14.0%)	172 (14.8%)	189 (16.2%)	182 (15.6%)	
Alcohol drinking	269 (23.6%)	316 (27.6%)	412 (35.9%)	435 (38.2%)	<0.001
Physical activity (MET)	51.3 (62.7)	70.7 (86.6)	100.5 (104.0)	126.9 (116.8)	<0.001
BMI (kg/m2)	23.1 (3.7)	23.4 (3.8)	23.1 (3.6)	22.6 (3.3)	<0.001
BMI>24 (kg/m2)	415 (38.3%)	461 (42.0%)	411 (37.8%)	320 (30.1%)	<0.001
Energy intake (kcal/d)	1714.0 (478.2)	2001.7 (520.6)	2206.8 (576.3)	2450.9 (676.1)	<0.001
Fat intake (g/d)	66.6 (34.9)	69.3 (36.3)	70.3 (38.3)	60.5 (36.6)	<0.001
Protein intake (g/d)	53.8 (19.0)	61.6 (20.5)	66.8 (23.0)	72.2 (25.2)	<0.001
Carbohydrate intake (g/d)	219.9 (55.6)	276.5 (69.1)	319.9 (82.3)	397.7 (116.8)	<0.001
Cumulative methionine intake (mg/d)	1192.6 (448.9)	1349.0 (373.5)	1418.8 (376.7)	1535.5 (358.3)	<0.001
Cumulative animal methionine intake (mg/d)	618.6 (431.3)	578.3 (373.4)	494.5 (377.9)	294.5 (317.2)	<0.001
Cumulative plant methionine intake (mg/d)	573.9 (101.3)	770.8 (41.6)	924.2 (52.3)	1241.0 (234.5)	<0.001
Most recent methionine intake(mg /d)	1197.0 (636.4)	1332.5 (484.2)	1411.9 (558.5)	1462.1 (526.6)	<0.001
Most recent animal methionine intake (mg /d)	649.8 (612.0)	617.6 (453.7)	554.6 (503.2)	332.3 (397.6)	<0.001
Most recent plant methionine intake (mg/d)	547.3 (131.0)	714.9 (147.2)	857.3 (190.5)	1129.7 (346.7)	<0.001
Lead intake (ug/d)	79.8 (25.0)	95.2 (24.9)	107.8 (29.0)	124.2 (39.2)	<0.001
Intake of fruit (g/d)	28.2 (71.4)	23.4 (75.9)	23.0 (78.5)	18.4 (91.3)	0.031
Intake of fresh vegetable (g/d)	216.5 (128.4)	261.6 (151.1)	298.0 (166.4)	323.8 (227.2)	<0.001
Intake of meat (g/d)	89.6 (81.2)	87.3 (81.0)	78.7 (91.9)	41.5 (63.5)	<0.001
Hypertension	456 (41.0%)	390 (34.9%)	401 (36.3%)	319 (29.5%)	<0.001
Diabetes	47 (4.1%)	52 (4.6%)	22 (1.9%)	28 (2.5%)	<0.001
Stroke	39 (3.4%)	22 (1.9%)	19 (1.7%)	20 (1.8%)	0.012
Self-reported poor memory	271 (23.5%)	229 (19.8%)	238 (20.6%)	226 (19.7%)	0.085

Self-reported memory decline	503 (44.4%)	424 (37.4%)	421 (37.0%)	431 (38.4%)	<0.001
Global cognition score	12.7 (6.9)	13.7 (6.5)	13.9 (6.6)	12.6 (6.8)	<0.001
Global cognition score < 7	248 (21.3%)	174 (14.9%)	168 (14.4%)	247 (21.2%)	<0.001

Supplement Table S2 Association between quartiles of methionine intake and cognition among Chinese adults.

	Quartiles of intake				
	Q1	Q2	Q3	Q4	P trend
Total methionine					
Global cognition score <7	1.00	0.99 (0.79-1.23)	1.07 (0.84-1.35)	**0.71 (0.53-0.95)**	0.100
Self-reported poor memory	1.00	0.94 (0.78-1.14)	0.86 (0.70-1.05)	**0.72 (0.57-0.92)**	0.007
Self-reported memory decline	1.00	0.97 (0.82-1.16)	0.85 (0.71-1.03)	**0.69 (0.56-0.85)**	<0.001
Animal methionine					
Global cognition score <7	1.00	**0.73 (0.59-0.91)**	**0.55 (0.43-0.71)**	**0.37 (0.27-0.50)**	<0.001
Self-reported poor memory	1.00	0.83 (0.68-1.01)	**0.72 (0.58-0.89)**	**0.60 (0.47-0.77)**	<0.001
Self-reported memory decline	1.00	0.86 (0.72-1.03)	**0.76 (0.63-0.92)**	**0.65 (0.52-0.80)**	<0.001
Plant methionine					
Global cognition score <7	1.00	**1.30 (1.02-1.66)**	**1.51 (1.16-1.97)**	**2.23 (1.63-3.03)**	<0.001
Self-reported poor memory	1.00	**1.31 (1.07-1.60)**	1.23 (0.99-1.54)	**1.38 (1.06-1.78)**	0.041
Self-reported memory decline	1.00	**1.19 (1.00-1.41)**	**1.21 (1.00-1.46)**	1.15 (0.92-1.43)	0.244

Values are odds ratio (95%CI) from mixed effect logistic regression.

Models adjusted for age, gender and energy intake, education, income, urbanization, smoking, alcohol drinking, and physical activity, intake of fruit and vegetable, BMI, hypertension, self-reported diabetes, and stroke.

All participants attended at least two waves of survey.
